# Temporal, spatial and household dynamics of Typhoid fever in Kasese district, Uganda

**DOI:** 10.1371/journal.pone.0214650

**Published:** 2019-04-22

**Authors:** Bernadette Basuta Mirembe, Stella Mazeri, Rebecca Callaby, Luke Nyakarahuka, Clovice Kankya, Adrian Muwonge

**Affiliations:** 1 Department of Biosecurity Ecosystems and Veterinary Public Health, Makerere University, Kampala, Uganda; 2 Division of Genetics and Genomics, The Roslin Institute, The Royal (Dick) School of Veterinary Studies, University of Edinburgh, Edinburgh, United Kingdom; 3 Participatory Epidemiological Network in Uganda (PENU), Kampala, Uganda; University of Tennessee Knoxville, UNITED STATES

## Abstract

Typhoid fever affects 21 million people globally, 1% of whom succumb to the disease. The social, economic and public health consequences of this disease disproportionately affect people in Africa and Asia. In order to design context specific prevention strategies, we need to holistically characterise outbreaks in these settings. In this study, we used retrospective data (2013–2016) at national and district level to characterise temporal and spatial dynamics of Typhoid fever outbreaks using time series and spatial analysis. We then selected cases matched with controls to investigate household socio-economic drivers using a conditional logistic regression model, and also developed a Typhoid fever outbreak-forecasting framework. The incidence rate of Typhoid fever at national and district level was ~ 160 and 60 cases per 100,000 persons per year, respectively, predominantly in urban areas. In Kasese district, Bwera sub-county registered the highest incidence rate, followed by Kisinga, Kitholhu and Nyakiyumbu sub-counties. The male-female case ratio at district level was at 1.68 and outbreaks occurred between the 20^th^ and 40^th^ week (May and October) each year following by seven weeks of precipitation. Our forecasting framework predicted outbreaks better at the district level rather than national. We identified a temporal window associated with Typhoid fever outbreaks in Kasese district, which is preceded by precipitation, flooding and displacement of people. We also observed that areas with high incidence of Typhoid fever also had high environmental contamination with limited water treatment. Taken together with the forecasting framework, this knowledge can inform the development of specific control and preparedness strategies at district and national level.

## Introduction

Typhoid fever is caused by *Salmonella* serovar Typhi and Paratyphi and affects approximately 21 million people annually of whom 222,000 succumb to the disease [1]. Furthermore, it has been estimated that 5.6 billion people are at risk globally [2]. Improving personal hygiene, environmental sanitation, food and water safety as well as limiting bacterial shedding by infectious individuals form the cornerstone of most control strategies for this disease [1]. Historically, development of good sewage management systems and water treatment has been credited for drastically reducing the incidence of enteric diseases in western Europe and North America in the 20^th^ Century [3]. The same trend is evident in Latin America and some Asian countries especially when compared with economic development which comes with improved access to water and sanitation [4]. Although there has been significant economic growth in Africa over the same period, the lack of significant investment in public health infrastructure means that the trends in disease incidence are not comparable with other parts of the world [5].

Countries like Uganda, with very high population growth rate and ample annual precipitation but with inadequate sanitation and safe water access, experience multiple large outbreaks of Typhoid fever every year [6, 7]. For example a recently documented Typhoid fever outbreak in the capital city Kampala was attributed to unconfined aquifers and registered 10,230 suspected cases, 1,920 of which were confirmed [8, 9].

Kasese is one of the districts with persistent outbreaks of Typhoid fever in Uganda [10]. Reports have suggested that some of the outbreaks can last as long as two years such as the one between 2007 and 2009. The outbreak spilled over into neighbouring districts like Bundibugyo and was detected after an influx in patients with intestinal perforations [11, 12]. The impact of such an outbreak causes large economic losses to already impoverished communities with approximately US$58 being spent on the treatment of an uncomplicated case, and going up to US$155 for those with intestinal perforations [13]. It is critical to understand the drivers of such Typhoid fever outbreaks at various levels in order to inform the development and implementation of targeted public health interventions. In this study we used retrospective data (2013–2016) at national and district level to examine temporal and spatial dynamics of Typhoid fever outbreaks. Furthermore, we selected cases and controls to investigate household socio-economic drivers of Typhoid fever and developed an outbreak-forecasting framework.

## Material and methods

### Study site

**Fig 1:**
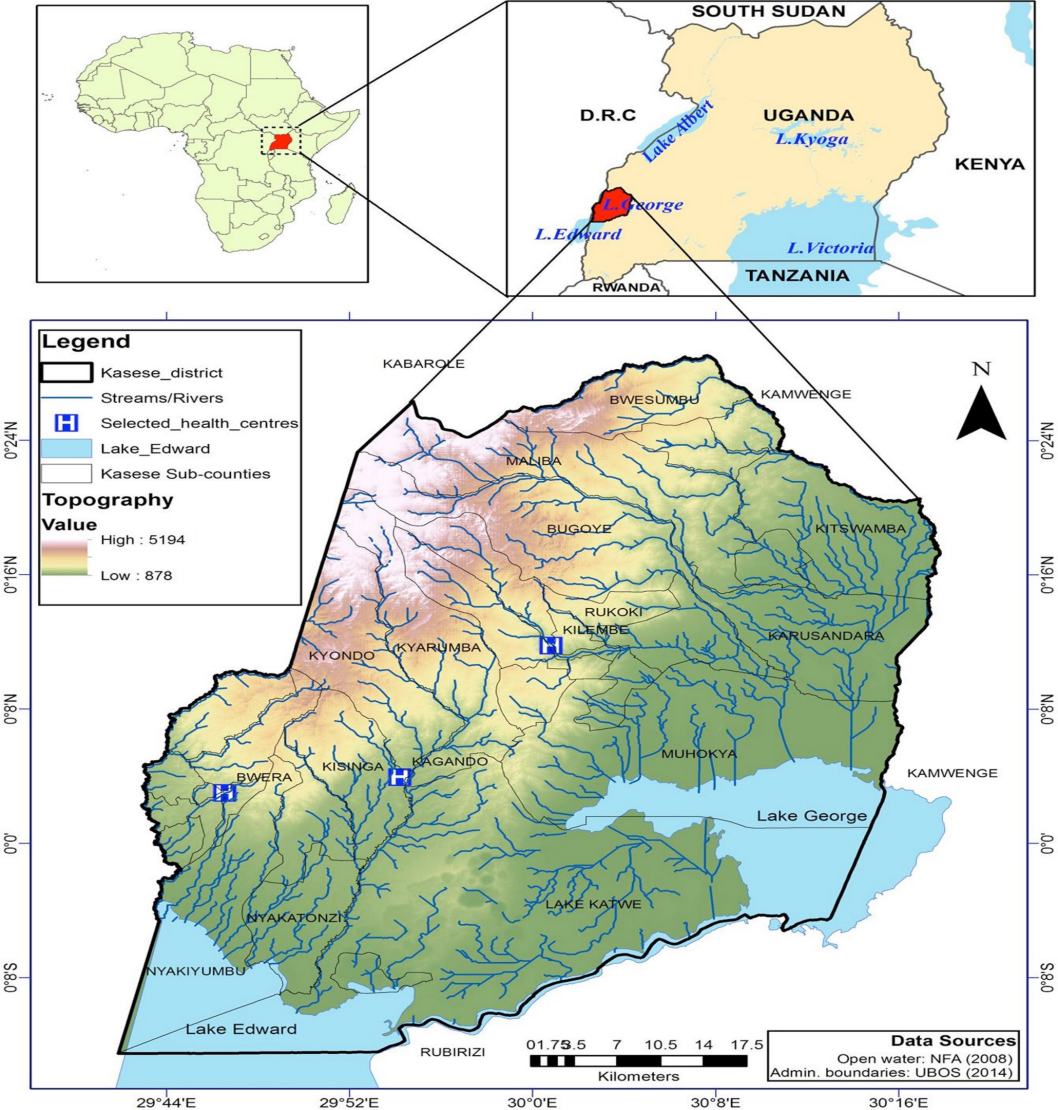
Shows the map of Kasese district, location of the three hospitals as well as rivers and lakes in this area. The colour gradient from brown to green represents the gradient in altitude in the district.

Uganda is a country in East Africa with a population of approximately 34.6 million people and population density of 173 people per km [14]. This study focused on Kasese district (Fig 1), which is located 371 kilometers west of the capital, Kampala. It covers a total surface area of 3,389.8km^2^, most of which is dry savannah gazetted for nature and wildlife conservation [15]. The annual average temperature and rainfall is 24.2°C and 1,424mm, respectively in two wet and dry seasons. Administratively, the district is subdivided into twenty-three sub-counties and is home to 702,029 people [16], of which 80% live in rural areas and 35% are under the age of 9. There are 139,406 households with an average household size of 5. The major economic activity (65%) in Kasese district is subsistence farming [14].

### Data sources

The work was based on three datasets representing three strata of the disease epidemiology linked to one another in time (2013–2016) and space (Kasese district in Uganda) (Figs A and B in S1 File). The first strata focused on the household dynamics of Typhoid disease. The second described the district level dynamics of the disease and focused on temporal and spatial trends in Typhoid fever. And the third examined the temporal trends of the disease at national level.

#### Hospital and household data

We got permission and reviewed Typhoid fever clinical records in Kasese district between 2013 and 2015 from three hospitals Kilembe mines, Bwera and Kagando. This data here formed the HOSPITAL_DATASET. Cases were defined as any individual who presented with Typhoid fever clinical signs i.e. fever, abdominal pain, nausea, diarrhoea/constipation, intestinal or abdominal discomfort including extended abdomen. They additionally had to have tests confirming *Salmonella* serovar Typhi/ Paratyphi infection by blood culture or *Salmonella* positive by a Widal test [17]. We got ethical consent to obtain the contact information of these patients, and with their consent enrolled them into the household level, case-control component of the study. This is referred to as HOUSEHOLD_DATASET in the study design section.

#### National level data

The Ministry of Health of Uganda hosts an online archive of weekly infectious disease outbreaks at district level [10]. This database contains weekly reports from more than 50% of the districts in Uganda, and is the most comprehensive data resource on infectious disease dynamics in the country [10]. We used national Typhoid fever data archived for Kasese district (NATIONAL_DATASET) to retrospectively characterise Typhoid fever outbreaks reported between 2013 and 2016.

#### Climate data

We also retrieved archived weather data in Kasese district via the Application Programming Interface (API) system for World Weather Online https://www.worldweatheronline.com[18]. Average weekly rainfall (mm), temperature (^0^C) and humidity (%) data between 2013–2016 was retrieved for this study.

### Study design and statistical analysis

#### Typhoid fever dynamics at the household level

We conducted a matched case-control survey for investigating the household level drivers of Typhoid fever. Cases were randomly selected from the HOSPITAL_DATASET i.e. every other case (i.e. skipping a case) starting from the last record in 2015 backward to the first record in 2013. Informed consent was obtained before a case was included in the household level study. Each case was matched to two controls geographically, by age and sex. The controls lived in the same residential area (village) as the case i.e. shared municipal amenities like water source, hospitals and markets. For controls to be selected they a) must have been resident in the same village as the case during the outbreaks; b) must not have been or had cases in their households and c) controls had to be consenting adults (≥18 years). Furthermore, regarding criterion b, we asked the control if they or any member of their household had had Typhoid fever within the last one-year period. A structured questionnaire was then administered to both cases and controls households to investigate personal hygiene, environmental sanitation, water and food safety practices and socio-economics in each of these households to generate the HOUSEHOLD_DATASET.

#### Sample size estimation

We visited 67 cases matched with 134 controls, providing us with a total sample size of 201 and a case to control ratio of 1:2. Assuming that there is a greater than 70% probability of developing Typhoid fever clinical signs once exposed to the causative agents then with this sample size we are able to detect odds ratio of 2.93 with 80% power and 95% confidence for environmental and household factors which are statistically associated with being a case *(**www*.*openepi*.*com*) [19].

#### Data and sample collection

In addition to questionnaire administration, environmental samples such as water from any source used by household, drinking water from storage containers within the household, faecal samples found improperly disposed within the compound of the household were collected for isolation of *Salmonella* species at the Central Diagnostic Laboratory, College of Veterinary Medicine, Animal Resources and Biosecurity, Makerere University. The isolated *Salmonella* species were used as an indicator of contamination and a proxy for sanitation at household level.

#### Household salmonella contamination

Isolation was done using a protocol developed by the FAO Manual of Food Quality Control: 4. Microbiology Analysis. FAO Food and Nutrition paper 14/4 [20]. The samples were placed in buffered peptone water then inoculated in Selenite broth and incubated at 42°C. Further inoculation of the samples on Xylose-Lysine Desoxycholate (XLD) agar after overnight incubation for direct plating to enable detection of *Salmonella* was done and incubated at 37°C. The suspected *Salmonella* spp were gram-stained and sub-cultured on nutrient agar (approximately 1–3 colonies). Biochemical tests (Indole, Methyl red, Simmon’s citrate, Urease and TSI) were performed to confirm presence of *Salmonella*. Disk-diffusion was used to measure susceptibility to selected antibiotics.

### Statistical analysis

#### Risk factors associated with typhoid fever

We developed a conditional logistic regression (CLR) model to identify associations between household and environmental factors of Typhoid fever using *clogit* function from the R package “survival” [21]. Since cases and controls were matched by geography, age and sex, the CLR considers matching of cases and controls in explaining the variation observed. Forward variable selection approach was used in model selection, where variables were added one at a time, starting with variables with the lowest p-value from the univariable analysis. Only variables with a p-value of less than 0.15 in the univariable analysis were considered for inclusion in the multivariable model. Upon addition of a new variable the Akaike Information Criterion (AIC) was calculated and the variable was kept in the model if the AIC was reduced. The least complex model was chosen based on the lowest AIC. To assess the fit of the model the Area Under the Curve (AUC) was calculated using the “ROCR” package [22].

#### Typhoid incidence in Kasese district

The incidence of Typhoid fever in each sub-county was calculated using the population demographic information obtained from Uganda 2014 National Census [16] and standard formulae for infectious diseases [23]. The incidence was mapped using the leaflet package in R [24] and shapefiles were obtained from OpenStreetMap and Uganda Bureau of Statistics [25, 26].

#### Typhoid fever dynamics at district level

We used a time series approach using the *forecast* library in R [27, 28] to identify temporal trends in the number of Typhoid fever cases at district and national level using the NATIONAL_DATA dataset. Missingness here means any week without data flanked by at least two weeks with data (unreported data within a potential outbreak). In this regard, 19/70 and 18/70 weeks were deemed missing for the national and Kasese district data respectively. There are several factors including logistics, extreme weather patterns, human error as well as technical communication breakdown cited a potential causes of missingness [29, 30]. Missing data was imputed using the “*imputeTS*” package [31]. Briefly, we generated a time-series variable (*ts*) from the number of cases reported between 2013 and 2016 at a frequency of 52 weeks per year. We used four imputation methods with interpolation and Kalman smoothing being the least and most conservative respectively[31]. Given that missing values were flanked by empirical data, we used the convergence of prediction across the methods as validation. We compared trends in the number of Typhoid fever cases observed at district level to the weather patterns using the cross-correlation function (*ccf*) in the forecast package in R [27, 28].

#### Forecasting typhoid fever dynamics in Uganda

In order to predict the number of cases in the first quarter of 2017 (January-April) for Kasese district and Uganda at large, we fitted the time-series variable generated in the previous section to an Autoregressive Integrated Moving Average (ARIMA) model using the ARIMA framework in the *forecast* package in R [27, 28]. The model fit was evaluated using the relevant post estimation methodology in this package including The Ljung-Box test statistic and its P-value as well as BIC and AIC.

### Ethical considerations

We sought IRB approval for the ethics of this study from Makerere University College of Veterinary Medicine, Animal resources and Biosecurity (SBLS/REC/16/140) and Uganda National Council of Science and Technology (A563). The District Health Office and Chief Administration granted permission to access patients’ records that was further endorsed by all the hospital directors. The participants were of consenting age (18 years and above) and signed a written consent agreeing to participate in the study. The signed consents were filed under lock and key. Data was coded and no person identifying information was used during data entry and analysis.

## Results

### Descriptive statistics of typhoid fever at the district and national level

**Fig 2:**
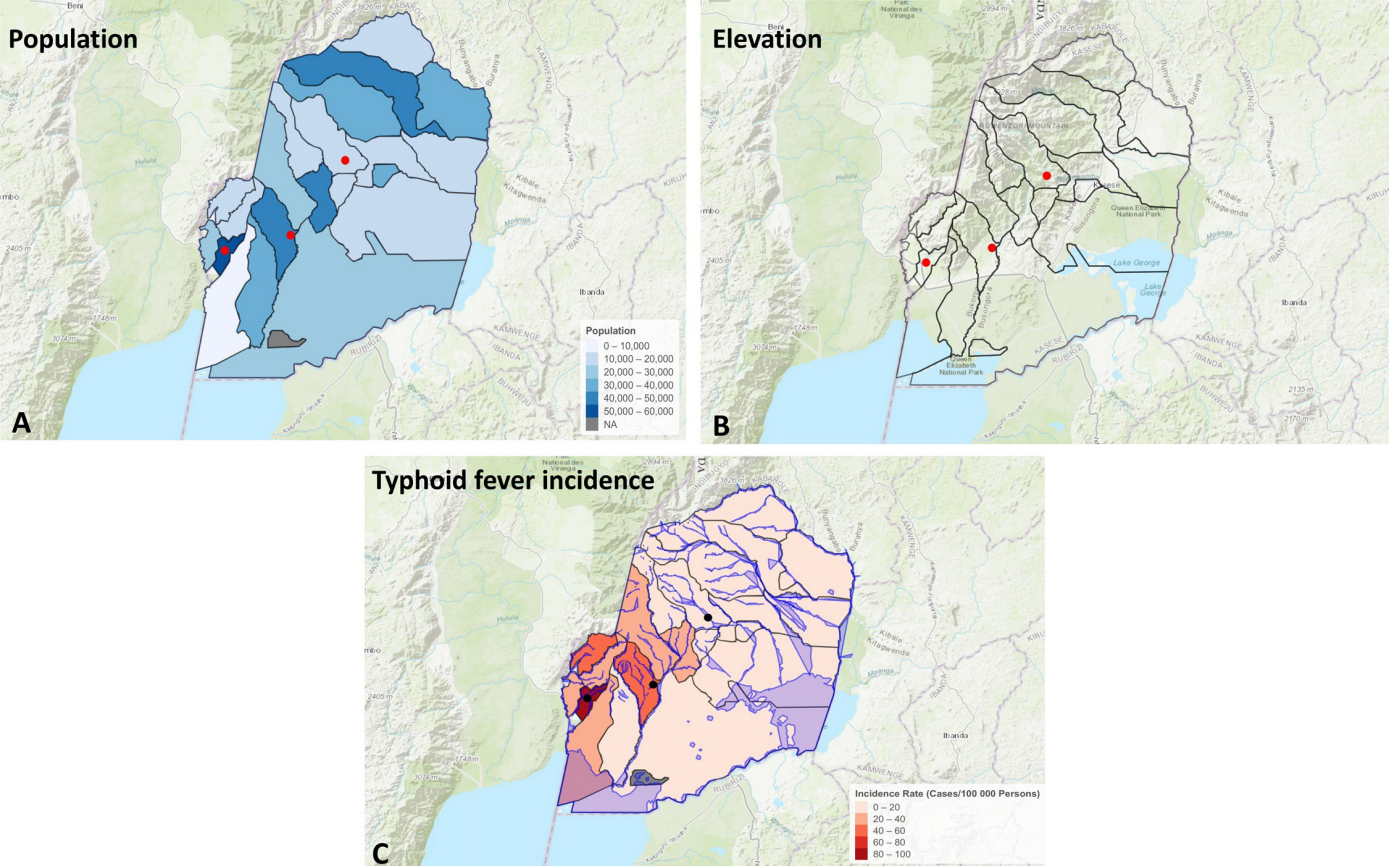
Shows incidence rate of typhoid fever cases per sub-county, population and elevation between 2013–2015 in Kasese district. The dots indicate location of the three hospitals where data used to compute the incidence rates was retrieved.

There were 210204 Typhoid fever cases reported in Uganda between 2013 and 2016, which gives an incident rate of approximately 160 cases per 100000 people per year. Majority of the cases reported at national level came from peri-urban central Uganda. During the same period, 1298 Typhoid fever cases were reported from Kasese giving the district a much lower incident rate of 60 cases per 100000 people per year. The retrospective dataset of Typhoid fever cases collated from three hospitals in Kasese district recovered information on 134 cases, majority of whom visited Bwera and Kagando hospitals. Bwera sub-county registered the highest incidence rate, distantly followed by Kisinga, Kitholhu and Nyakiyumbu sub-counties (Fig 2). In the HOSPITAL_DATASET, the clinical profile showed a male: female case ratio of 1.68 and most of the cases were residents of urban and peri-urban areas of the district. The median age bracket of cases was 20–40, and we also observed that ~ 70% of the cases were between 18–40 years of age.

### Typhoid fever dynamics at district and national level

#### Temporal and spatial characteristics of typhoid fever

**Fig 3:**
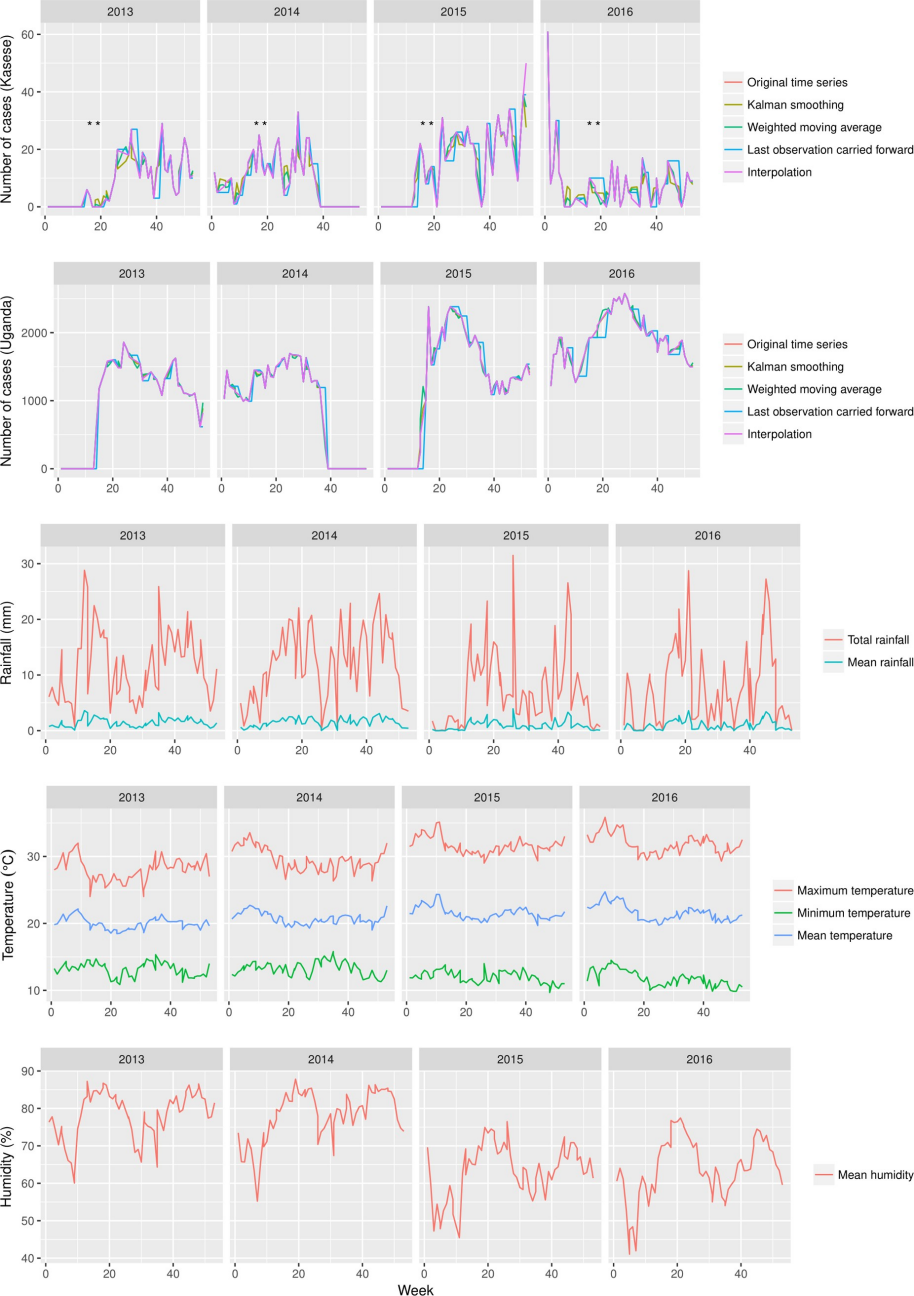
Shows the cases of typhoid fever at National and Kasese district level as reported on at weekly basis in the Ministry of health National surveillance report between 2013 and 2015 (n = 126,205 and 1298 respectively). The dotted line in all the three panels of this figure shows the moving average. The third panel in this shows the rainfall distribution in Kasese district, highlighting weeks of flooding in each year [32–35]. In this regard, the first flooding in week 16 of 2013 was followed by displacement of 9663 people of who 5072 were moved to internally displaced camps in Bulembiya [32, 33]. The second flooding in week 19 of 2014 was followed by displacement of 603 people. The third and fourth flooding in week 17–21 & 27 of 2015 were followed by ~7000 people [32, 34, 35]. The x-axis represents time in weeks, A-C represents weeks in 2013–2015 respectively.

The comparative temporal dynamics at national and district level are presented in Fig 3. The trend shows that each year Kasese district experiences Typhoid fever outbreaks between weeks 20 and 40. We observed other outbreaks before and after this twenty-week window. For example in 2013, an outbreak occurred between weeks 40 and 52 and the outbreak at the end of 2015 spilled over into January 2016. The subsequent outbreaks in 2016 were small in size but occurred throughout the year. In general, the trends at national level mirror those observed in Kasese district. In contrast, the largest outbreak at national level was observed in mid-2016. When we compared the trends between cases and the weather pattern in Kasese district, we observed that the spike in Typhoid fever cases in June and October 2014 coincided with precipitation and increased humidity. In contrast, the spike in Typhoid fever cases in July and October 2015 did not coincide with precipitation and humidity and the same was true for the spike at the end of that year. Indeed, the cross-correlation analysis between Typhoid fever cases and precipitation showed a negative correlation with a 0.15 (7 weeks) lag between precipitation and case reporting. The relationship with temperature cyclic i.e. both positively and negatively correlated with lags before and after case reporting (Figs C and D in S1 File**).**

There was a considerable difference in the annual incidence profile of Typhoid fever in Kasese district at the three altitude levels (Low<1180, Medium 1180> x <1382 and High>1382 meter above sea level). In general, most of the cases (56%) were residents of low altitude settings.

### Household level dynamics of typhoid fever

The case control survey gathered information from 201 households, with an average household membership of 7.45, therefore a population of ~ 1500 person was investigated. The median age of cases and controls was 36 years (range 18–84). It is worth noting that the “adult consenting age” was one of our selection criteria (Figs E and F in S1 File).

#### Salmonella *spp* contamination at household level

The findings in Table A in S1 File show the household samples from which Salmonella was recovered. We recovered *Salmonella enteritidis* from samples collected from both case and control household samples. There was no statistical difference in the levels detected between these two groups although the proportion of drinking water samples from which *Salmonella* was detected in case households was almost double that of controls (Table A in S1 File).

#### Multivariable association of typhoid fever cases with household factors

The conditional multivariable logistic regression model in Table 1 shows the domestic and sociological factors associated with being a Typhoid fever case in Kasese district. Drinking water treatment practices was one of the factors associated with being a case i.e.; the odds of being a case was 9.23 for those who treated drinking water compared to those who did not. Washing hands with soap was more likely to be among cases than controls, this however was only marginally significant in our model. The level of education was not significantly associated to being a case.

**Table 1:** Conditional multivariable logistic regression model showing the association between household attributes and being a Typhoid fever case in Kasese district.

Variable	Level	Cases	Controls	P-value	Adjusted OR (95%CI)
Level of formal education	None	8	40	Ref	1
	Primary	33	63	0.14	2.02(0.79–5.13)
	Secondary	19	19	0.91	1.07(0.34–3.36)
Do you treat your drinking water?	No	48	104	Ref	1
	Yes	19	30	>0.001	9.23(4.84–17.61)
Do you wash hands with soap	No	19	59	Ref	1
	Yes	48	73	0.07	1.81(0.95–3.45)

AIC 253.517, Concordance = 0.931

### Forecasting typhoid fever dynamics at district and national level

**Fig 4:**
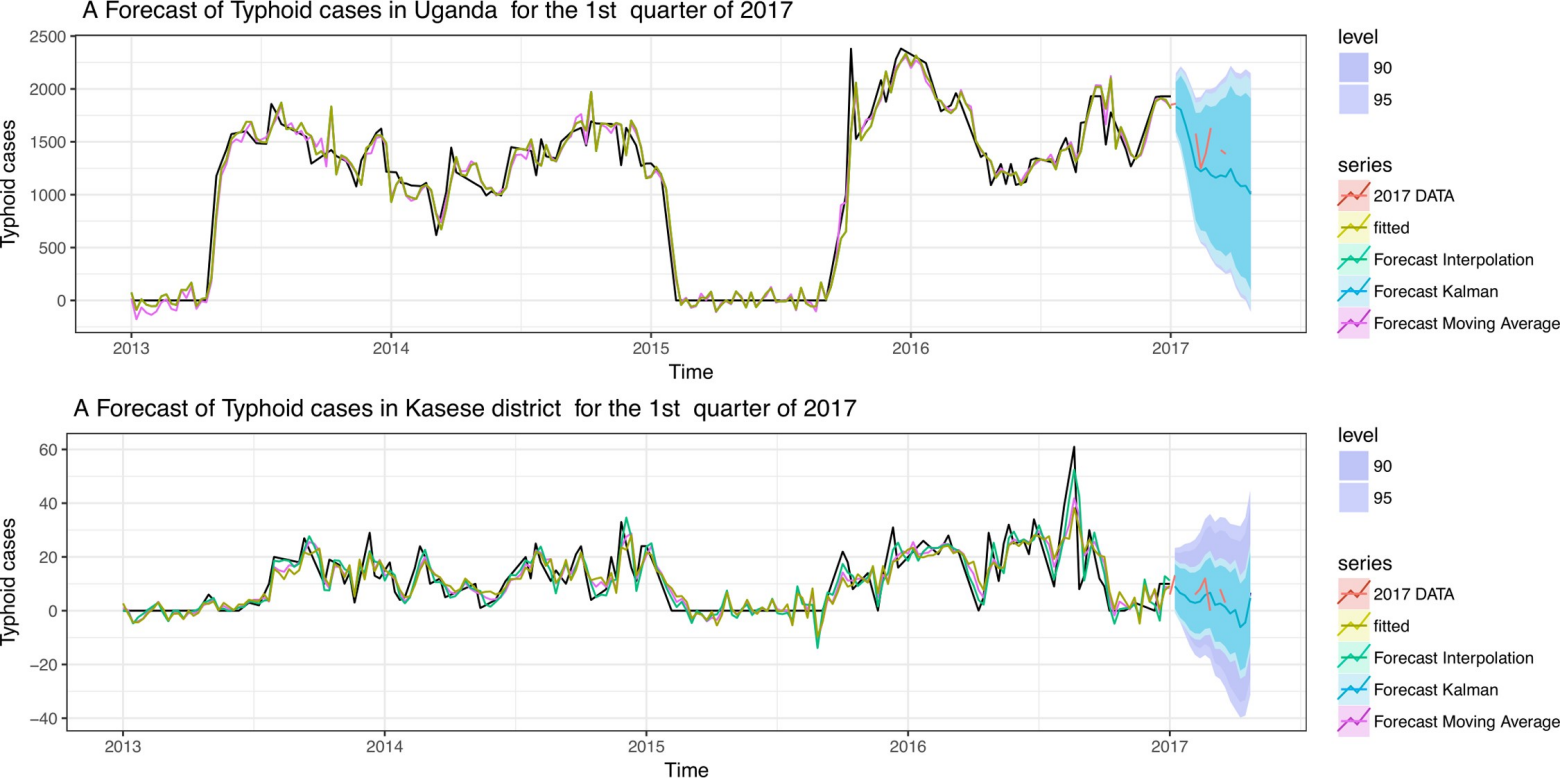
Shows the results of the typhoid fever outbreak-forecasting framework for the first quarter of 2017 at national and Kasese district level.

Fig 4 shows our fitted ARIMA model on the four-year’s worth of Typhoid fever incidence data (2013–2016). We have used it to recapitulate the temporal trends for the first quarter of 2017 at national and district level with the forecasting framework. Note that the model selected had the lowest complexity AIC = 1491.77 & 1799.12, p-value = 0.59 & 0.98 for Kasese district and national level respectively (See Figs G and H in S1 File), this model reasonably predicted the caseload as well as the general trend of the first quarter of 2017 with variable levels of uncertainty. We observed slightly better prediction at district level than at National level. Note that the p-value here indicated that there is a difference between what the model predicts and what the dataset’s dynamics represent.

## Discussion

The public health burden of diseases like Typhoid fever disproportionately affects people in developing countries especially in Africa where the population is projected to hit 2.5 billion by the year 2050 [36, 37]. Such countries are also characterised by scarcity of epidemiological data which not only limits our ability to implement robust and targeted control strategies, but also the attainment of health related Sustainable Development Goals (SDGs) [36]. In light of the SDGs, it is critical now more than ever to understand outbreaks at all levels and to document context specific drivers in the most affected populations. This seems to be the only cost-effective way to guide the development and implementation of public health interventions. In this study, we used retrospective data at national and district level to examine temporal and spatial dynamics of Typhoid fever outbreaks. Furthermore, we selected cases and controls to investigate household socio-economic drivers in addition to developing Typhoid fever outbreak forecasting models. This multi-method analysis at different administrative levels of the community provides breadth and depth for developing robust strategies that limit the risk of exposure and impact of Typhoid fever on communities.

### Descriptive summary of typhoid fever

There were 210,204 Typhoid fever cases reported at national level between 2013 and 2016 i.e. ~ 160.1 cases per 100,000 people per year. This estimate not only falls within the range previously reported [10] but also supports the continental ranking that puts Uganda, Djibouti, DRC and Tanzania on top [38, 39]. In economic terms, even if we took the most conservative disease management estimates as reported by [13], i.e. outpatients without intestinal perforations (IP), the total treatment cost would be ~ $7 million at national level. On the other hand, if we take the estimates for IP and none IP outpatients in Kasese district in this period, the total disease management cost would be ~ $110,000 and $301,676 respectively. This is an enormous financial burden placed on individuals in communities mostly struggling with people displacement and property destruction due to the heavy rains[32–35].

Most of the cases in Uganda are predominantly reported in peri-urban settings [9]. Such settings are characterised by poor personal hygiene, environmental sanitation and limited supply of municipal water, all of which are critical risk factors for Typhoid fever [40]. It therefore not surprising that the most affected populations in the region are slum dwellers [38, 41]. Over the years, there has been a considerable improvement in Typhoid fever surveillance at national level, which is partly due to efforts by Typhoid Surveillance in Africa Programme (TSAP) [42]. These efforts have however tended to lack granularity about individual outbreaks which makes it difficult to learn from the outbreaks. In this study, we observed that at district level in Kasese, the case distribution was skewed towards the male gender. This gender skew has also been reported elsewhere [11, 12], where it was believed to reflect the disparity in health seeking behaviour between males and females. However, previous studies conducted on gender health seeking behaviour in Uganda have consistently shown that females were better at seeking medical attention than males [43, 44]. The observed gender case distribution is therefore likely an indication of intersectionality between masculine roles or activities and the Typhoid fever risk profile.

### Temporal and spatial dynamics of typhoid fever

A general lack of robust diagnostic capacity in developing countries means that accurate temporal characterisation of gastro-intestinal infections is difficult [45]. Therefore, characterising temporal signatures of Typhoid fever, which can serve as diagnostic correlates within clinical diagnostic algorithms is very essential in these settings. For example, we observed an annual incidence between week 20 and 40 (May and October). Our cross-correlation analysis between weather parameters and cases reported in Kasese district reveal 0.15 (7 weeks) lag between precipitation and case reporting i.e.; an outbreak peak was preceded by a bout of precipitation. On the other hand, this relationship is cyclic for humidity and temperature i.e. a lag before and after onset of cases. It is worth noting that this lag between these weather parameters and onset of disease will be shorter. Such information has potential to improve the efficiency of diagnostic triaging at clinical level in specific hotspot areas of the country. Water is a central factor to Typhoid fever outbreaks [45], which means that sustained precipitation in settings devoid of efficient drainage systems and water treatment presents considerable risk to its dwellers. Indeed, most reports of Typhoid fever outbreaks have shown a close association between precipitation and outbreaks [46, 47]. In Dhaka for example, the Typhoid fever risk was linked to distance of a case from a water body and river water levels, suggesting that flooding played a role in pathogen dissemination [47].

Kasese is one of the most flood prone districts in Uganda; in fact this district has experienced flooding in April and May of every year since 2013 [33]. The banks of rivers Nyamwamba, Mubuku and Nyamugasani burst inundated six sub counties i.e. Bulembia, Karusandura, Kyarumba, Nyamwamba and Central division along their paths to Lake Edward and George [32, 34, 35]. These floods have displaced up to 40,000 people in the last four years [33]. If we put this in the temporal context characterised in this study, it means that the flooding and displacement of people usually occurs between the 15^th^ and 22^nd^ week of each year which is ~ 5 weeks before the onset of high Typhoid fever case reporting.

We observed the highest incidence in Bwera among other sub-counties. Bwera sub-county is predominantly a low altitude urban area with a high population density. Dwellers of Bwera sub-county use municipal water predominantly pumped from rivers and protected springs [48]. This means that a significant proportion of the population is reliant on water sources whose safety is known to fluctuate with environmental contamination [49, 50]. It is therefore more likely that the floods, which follow weeks of heavy precipitation lead to contamination of water sources that potentially overwhelm any water treatment strategies. This would compromise the safety of water available for communities undergoing varying levels of displacement.

Kisinga, Kagando, Kyondo and Kyarumba sub-counties also had a high incidence of Typhoid fever cases, these sub-counties are close to the main tributaries of River Nyamugasani with the Kisinga and Kagando laying downstream. Literature indicates that a large proportion of the populations living in these two areas have limited access to safe water [48]. One can therefore argue that the Typhoid fever risk for these counties were likely modulated by the a) levels of environmental contamination resulting from flooding and b) that outbreaks in Kyondo and Kyarumba upstream of the river Nyamugasani likely play a role in the outbreaks that occur downstream in Kisinga and Kagando. Although this study cannot make this link empirically, the temporal dynamics show that between March and June 2015, the case incidence in Kyondo and Kyarumba was preceded by higher case incidence in Kisinga and Kagando (Fig 1).

### Household socio-economic drivers of typhoid fever in Kasese

In most sub Saharan countries, the household is the primary unit of interaction, therefore understanding factors associated with being a Typhoid fever case at this level is the cornerstone for any control strategy. We started by examining sanitation; where we used *Salmonella* contamination as a microbial proxy for sanitation. The findings do not show any salient difference in *Salmonella* spp contamination between samples collected from households of cases and controls except for drinking water samples. Although not statistically significant, we observed that the proportion of drinking water samples from which *Salmonella* species were isolated was twice as high in cases. Indeed some studies have shown that such characteristics can be an integral part of the household Typhoid fever risk profile [51]. For example, only 32.8% of a community in Malawi was found to treat water after it had suffered an outbreak [52]. Microbial quality of drinking water deteriorates during storage at household level [53, 54] usually as a function of how water storage receptacles are handled. This could explain why *Salmonella spp* were more prevalent in case households water. In general, sanitation is a product of routine behavioral activities, which in combination with other household, spatial and temporal factors ultimately determined who became a case in a given Typhoid fever outbreak. For example, the conditional logistic regression model shows that water treatment practices were associated with being a case i.e. we found that the odds of being a case were 9.23 higher in those who treated drinking water compared to those who did not treat drinking water. This seems counter intuitive and in disagreement with most studies which have reported water treatment to reduce microbial contamination significantly thus protective of disease [55]. In this study we visited cases months after treatment, as part of their treatment cases are also sensitized on hygiene and water treatment to avoid future infections. We believe this could explain why more cases appear to treat drinking water than controls. Some studies have also shown that despite proper water treatment, microbial quality of water deteriorates during storage due to handling [56–58]. It is therefore likely that case-household re-contaminated water during storage after treatment; this could explain why we found case households having twice the level of contamination in drinking water when compared to controls. Therefore, adherence to water treatment is critical in such situations [59–61]. This is usually compromised by the complacency due to the belief that clear tap water is safe for consumption in urban settings.

Most studies have shown age as key factor in driving the risk profile of Typhoid fever in communities [11, 38] and one that can be exploited for targeted control [38, 45]. In this study however, because age was part of the selection criterion for cases, there is an inherent bias in the age distribution profile. None the less, our case distribution by age is similar to what was recently reported in a 10 year study conducted in China [62].

### Temporal forecasting for typhoid fever outbreaks

Temporal predictions of infectious disease outbreaks can play a central role in informing preparedness and control strategies [63] i.e. surveillance, vaccination and routine clinical planning [64]. In this study, the forecasting framework that we developed reasonably predicts the spikes in case reporting which we consider as outbreaks in the first quarter of the year 2017 at district level, specifically in the 9^th^ week (February-March). Most importantly we were able to forecast the trend in the first quarter both at the district and national level. Such information is not only important for national preparedness in terms of medical consumables to stock but also for allocating scarce resource for districts that are most likely to be adversely affected. There is room to improve the robustness of such models especially as more data is collected through surveillance as shown in a recent comparative study in China [64].

### Public health perspective

A multi-level approach to understanding the dynamics of Typhoid fever outbreaks provides a holistic tool kit to develop and implement synergistic and complementary cost effect ways of prevent spread and or avert socio-economic impact. For example; we used a) a time series analysis which identified annual risk periods between the 20^th^-40^th^ week of each year (May to October), b) and a recorded high incidence of Typhoid fever in Bwera, Kisinga, Kagando Kyarumba and Kilembe sub-counties, c) we showed that incidence of these outbreaks is preceded by seven weeks of heavy precipitation occurring between the 15^th^-20^th^ week (April-May) which are usually followed by people displacement due to flooding. It is important to note the onset of disease is likely much early; and that we used reporting which does not occur in real-time. By including a case-control study, we also identified household factors such as water treatment and hygiene practices as well as contamination levels. Such a multi-level risk profile can guide preparedness and also facilitate the allocation of scarce resources at district and national level in Uganda. Furthermore, the spatial, temporal and household risk parameters could be exploited to improved clinical diagnostic algorithms at district level.

### Limitations of the study

We were able to recover only 10% (134/1298) of the national level records at the three hospitals in Kasese. None the less we were able to recruit a sufficient number of cases and controls to allow for a statistically robust association analysis.

## Conclusions

Exploiting temporal, spatial and household data at various administrative levels using time series allowed us to characterise Typhoid fever outbreaks with more granularity in Kasese district. We identified a temporal window that is consistently associated with Typhoid fever outbreaks; precipitation, flooding and displacement of people precede this window. Furthermore, these outbreaks were mainly located in a river basin. We also observed that cases are associated with salmonella contamination of drinking water receptacles at household level. In this regard, we have developed a forecasting framework whose utility can be better harnessed at national level. Taken together, this output can play a central role in developing context specific control and preparedness strategies at national and district level.

## Supporting information

S1 File    •  **Fig A: The referral system in Uganda** shown by solid arrows while the broken arrow shows the typhoid surveillance and reporting.
   •  **Fig B: The case selection process from surveillance and archived data at national and district level respectively**.
   •  **Fig C and D: shows the cross correlation output between the typhoid case incidence and temperature and rainfall respectively.** The dotted blue line shows the level above or below which the autocorrelation function is statistically significant. A spike that is below or above this corresponds with a lag at which the two time series are significantly correlated. Prevalence of cases with and without intestinal perforations analysis is based on clinical data in HOSPITAL DATASET. In general, there is a high caseload between April and July and again between October and November. Indeed majority of the cases present with intestinal perforations across the year.
   •  **Fig E: A density plot (equivalent to the proportion cases and controls) for a given number of household members (I) and age of the respondent (II).** 0 and 1 represented in pink and light blue colour correspond to controls and cases respectively. There is considerable overlap between the two curves therefore we are confident there was no significant difference in the two groups on these two aspects. Note that the same relationship is true for sex of the cases and controls.
   •  **Fig F: The spatial matching of cases and controls in Kasese district**.
   •  **Fig G: The model validation for the National Typhoid fever time series model between 2013–2016.** The autocorrelation plot shows that we have four spikes that cross the levels of significance. The Ljung-Box test statistic is 73.2, and the p-value is 0.98. There for there is little evidence of non-zero autocorrelations in case incidence forecast at lags 1-.
   •  **Fig H: The model validation for the Kasese Typhoid fever time series model between 2013–2016.** The autocorrelation plot shows that we have four spikes that cross the levels of significance. The Ljung-Box test statistic is 97, and the p-value is 0.59. There for there is little evidence of non-zero autocorrelations in case incidence forecast at lags 1–70.
   •  **Table A: A univariable exploration of variable associations with being a Typhoid fever case in Kasese district.** The characteristics of all variables explored in this study are presented in this table that is divided into seven sections i.e.; demographic characteristics, environmental water sources (usually communal), individual handling of drinking water in household, household hygiene, household food safety, household sanitation and household contamination with Salmonella spp.
   •  **Table B: The case predictions from the time series forecasting framework and empirical date at national and district level for the first quarter of 2017.** Model for national forecasting AIC = 1491.77 and BIC = 1515.13, Model for district forecasting AIC and BIC for Kasese and National framework of 1799.12,1809.14 and 3189.76, 3199.79.
   •  **Text A: Questionnaire used in the case-control study.**
   •  **Text B: Consent form used in the case control study.**
(PDF)
