# Supplementary material for: Temporal, spatial and household dynamics of Typhoid fever in Kasese district, Uganda
Source: PLoS One. 2019 Apr 22;14(4):e0214650. doi: 10.1371/journal.pone.0214650 (PMC6476469; doi:10.1371/journal.pone.0214650)
Supplement: S1 File — • Fig A: The referral system in Uganda shown by solid arrows while the broken arrow shows the typhoid surveillance and reporting. • Fig B: The case selection process from surveillance and archived data at national and district level respectively. • Fig C and D: shows the cross correlation output between the typhoid case incidence and temperature and rainfall respectively. The dotted blue line shows the level above or below which the autocorrelation function is statistically significant. A spike that is below or above this corresponds with a lag at which the two time series are significantly correlated. Prevalence of cases with and without intestinal perforations analysis is based on clinical data in HOSPITAL DATASET. In general, there is a high caseload between April and July and again between October and November. Indeed majority of the cases present with intestinal perforations across the year. • Fig E: A density plot (equivalent to the proportion cases and controls) for a given number of household members (I) and age of the respondent (II). 0 and 1 represented in pink and light blue colour correspond to controls and cases respectively. There is considerable overlap between the two curves therefore we are confident there was no significant difference in the two groups on these two aspects. Note that the same relationship is true for sex of the cases and controls. • Fig F: The spatial matching of cases and controls in Kasese district. • Fig G: The model validation for the National Typhoid fever time series model between 2013–2016. The autocorrelation plot shows that we have four spikes that cross the levels of significance. The Ljung-Box test statistic is 73.2, and the p-value is 0.98. There for there is little evidence of non-zero autocorrelations in case incidence forecast at lags 1-. • Fig H: The model validation for the Kasese Typhoid fever time series model between 2013–2016. The autocorrelation plot shows that we have four spikes that cross the levels of sign [file pone.0214650.s001.pdf]

Supporting information

Temporal, spatial and household dynamics of Typhoid fever in Kasese district, Uganda

Bernadette Basuta Mirembe[1*] Stella Mazeri[3], Rebecca Callaby[3] Luke Nyakarahuka[1] Clovice Kankya[1, 2] Adrian Muwonge[1, 3]

[1] Department of Biosecurity Ecosystems and Veterinary Public Health, Makerere University, Kampala, Uganda

[2] Participatory Epidemiological Network in Uganda (PENU), Kampala, Uganda

[3] Division of Genetics and Genomics, The Roslin Institute, The Royal (Dick) School of Veterinary Studies, University of Edinburgh, UK Easter Bush Campus, EH259RG, Edinburgh, United Kingdom

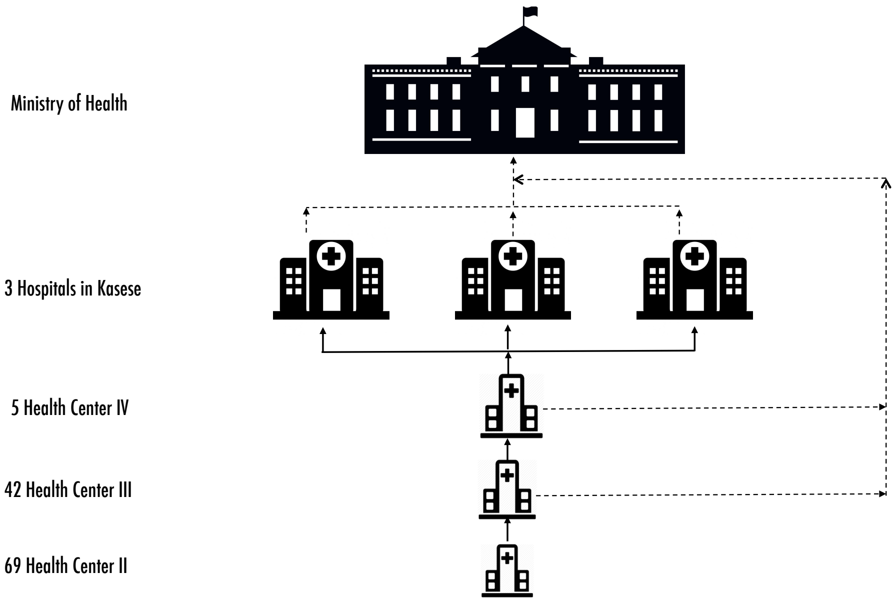

Fig A: The referral system in Uganda shown by solid arrows while the broken arrow shows the typhoid surveillance and reporting

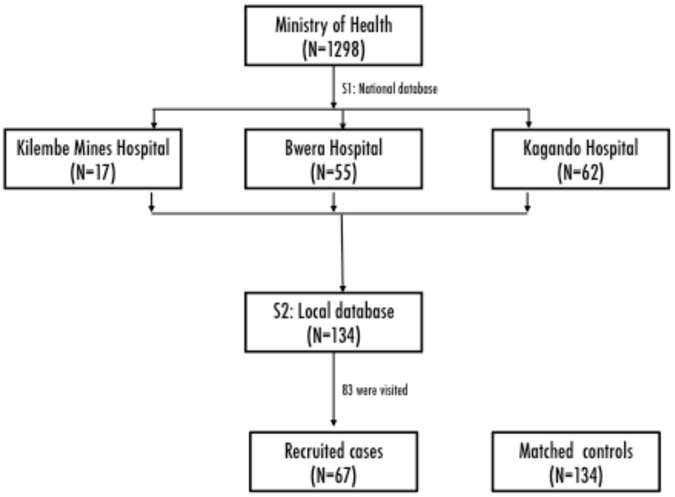

Fig B: Shows the case selection process from the surveillance and archived data at national and district level respectively

Cross correlation analysis of typhoid incidence and weather pattern

C

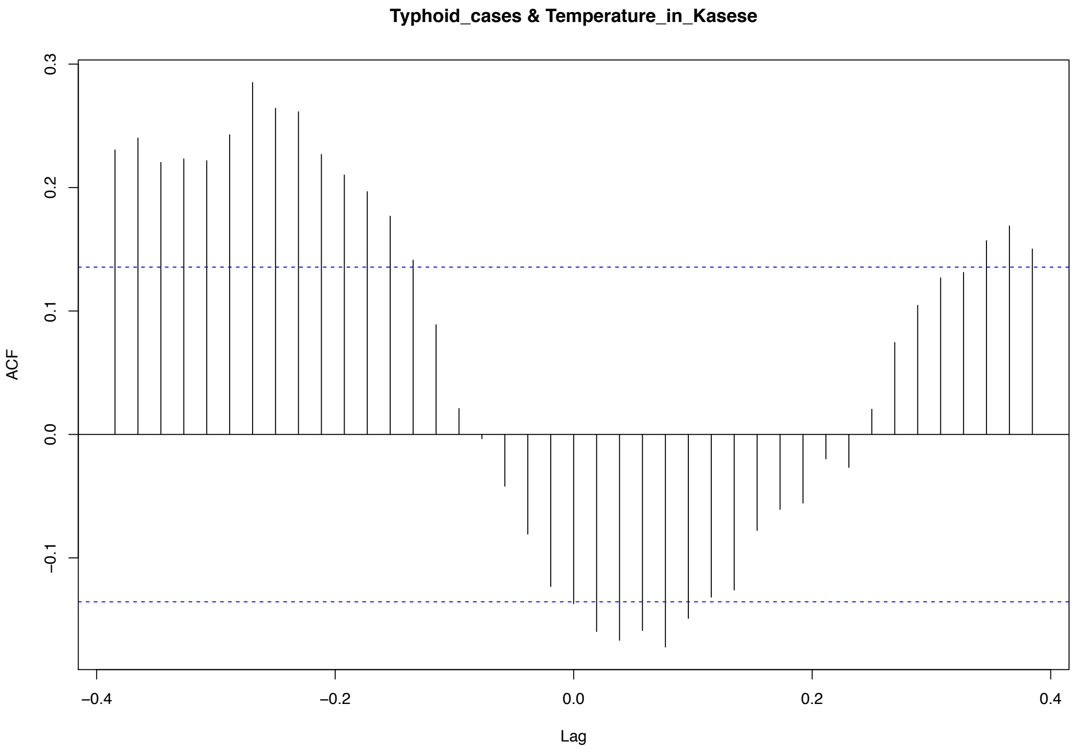

D

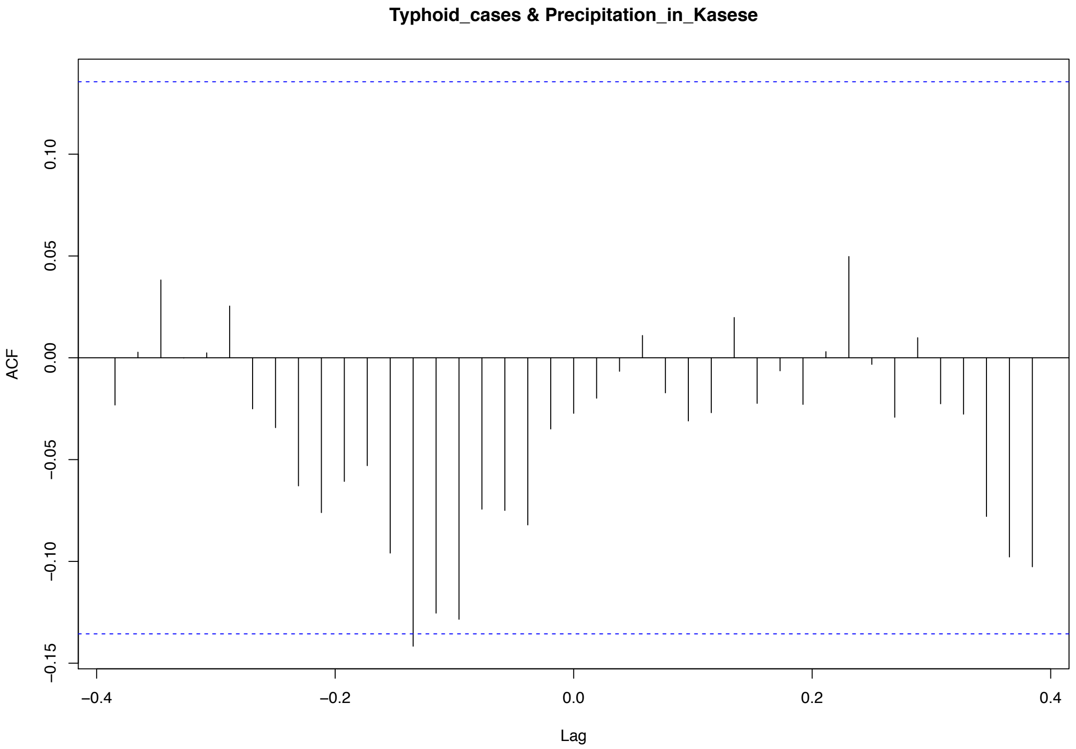

Fig C and D shows the cross correlation output between the typhoid case incidence and temperature and Rainfall respectively. The dotted blue line shows the level above or below which the autocorrelation function is statistically significant. A spike that is below or above this corresponds with a lag at which the two time series are significantly correlated.

Case and control Matching

I **II**

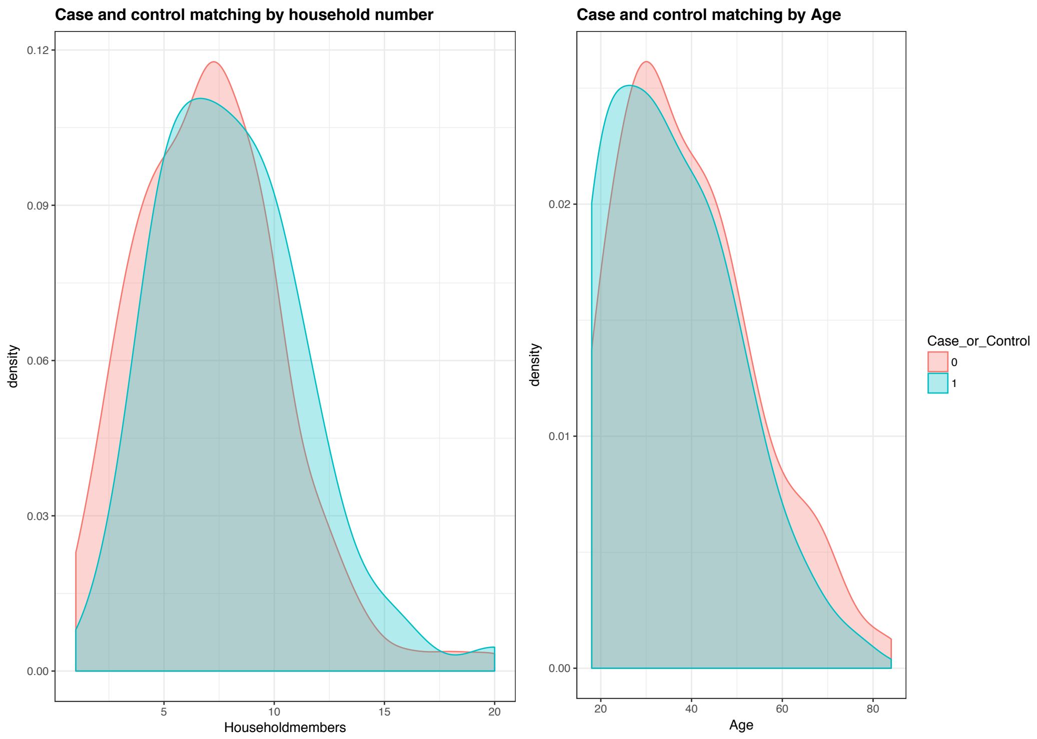

Fig E shows a density plot (equivalent to the proportion cases and controls) for a given number of household members (I) and age of the respondent (II). 0 and 1 represented in pink and light blue colour correspond to controls and cases respectively.

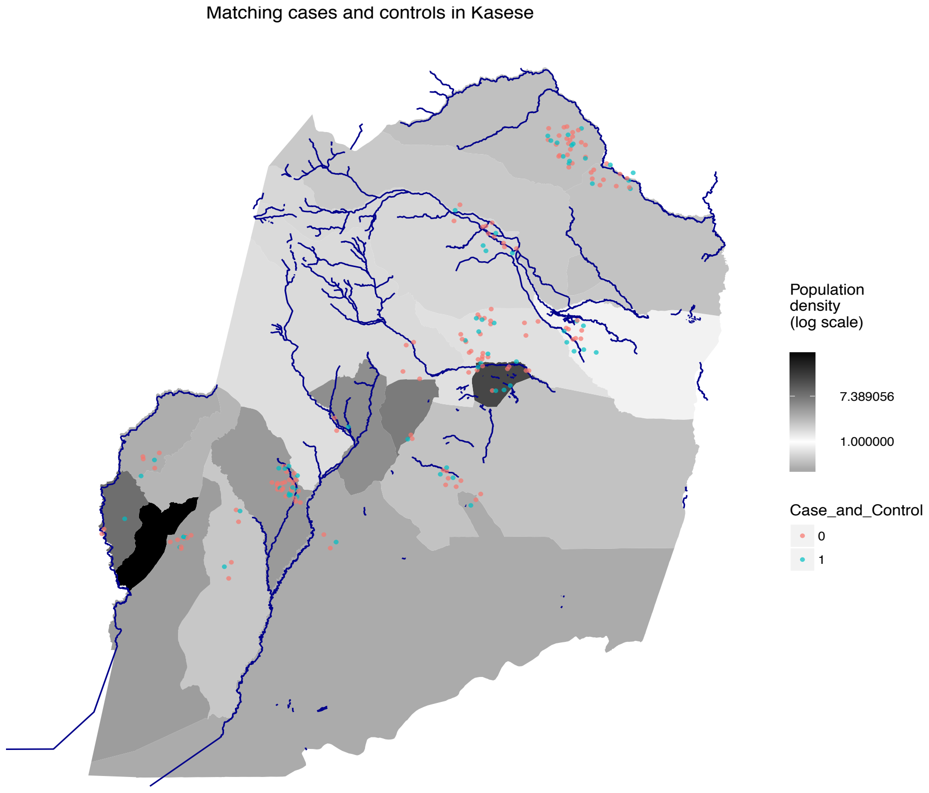

Fig F: Shows the spatial matching of cases and controls in Kasese district

A Forecast model fitting with Arima

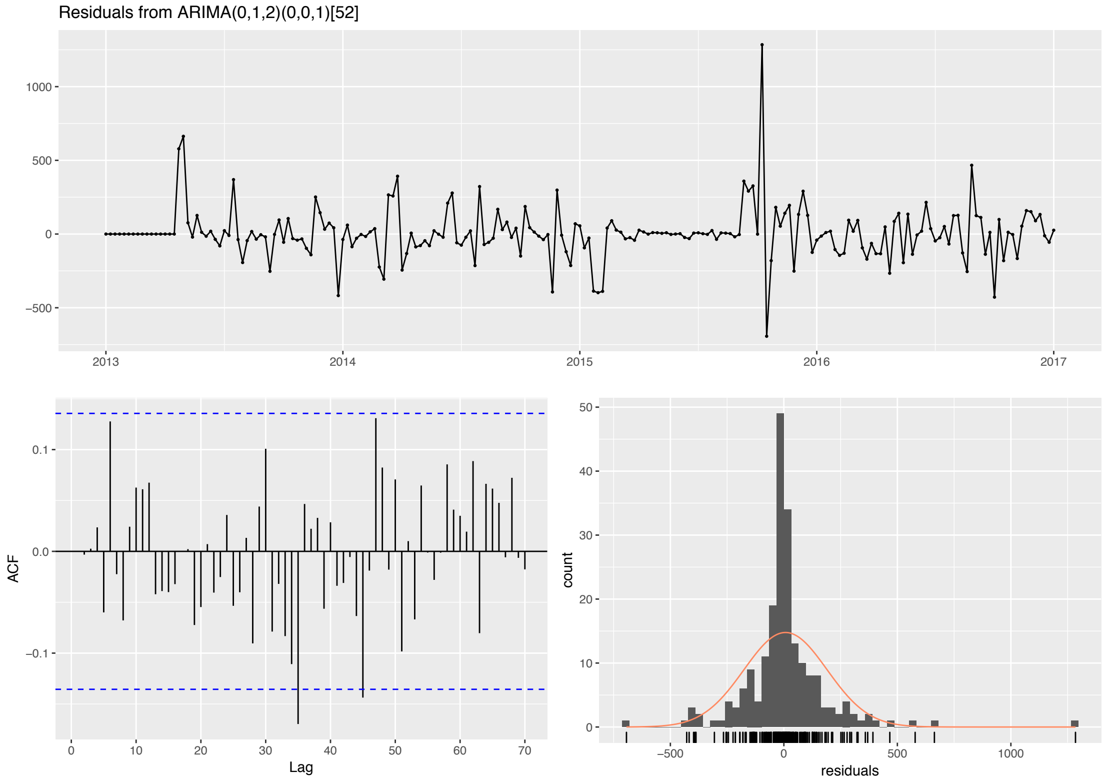

Fig G shows the model validation for the national typhoid time series model between 2013-2016.

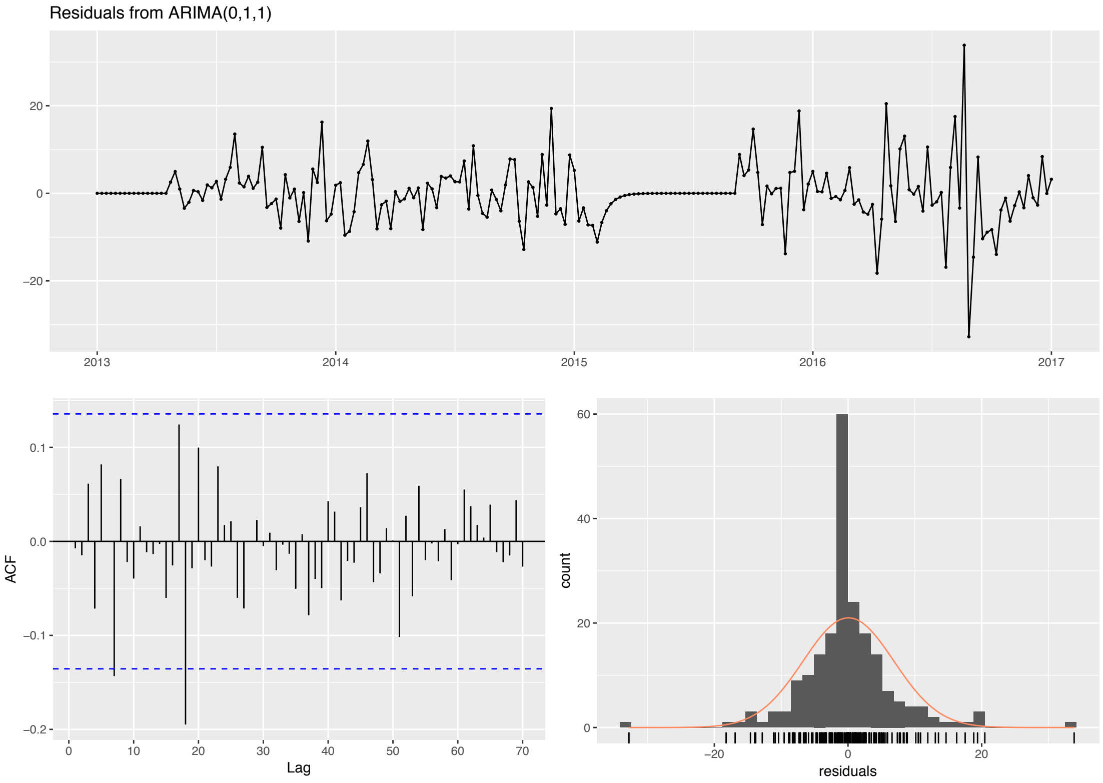

Fig H shows the model validation for the Kasese typhoid time series model between 2013-2016.

Table A: A univariate exploration of variable associations with being a typhoid case in Kasese district

Variable	Response	Proportion of Cases (%)	Proportion of Controls (%)	P-value	Unadjusted OR (95% CI)
**Gender of Respondent**	Male	25(37.3)	41(30.6)	Ref	Ref
	Female	42(62.6)	93(69.4)	0.339	0.74(0.40-1.37)
Age of Respondent	>=45	18(26.8)	46(34.3)	Ref	Ref
	31-44	20(29.8)	41(30.5)	0.211	1.24(0.58-2.69)
	18-30	29(43.2)	47(35.0)	0.576	1.57(0.77 3.26)
Respondent's education level	None	8(11.9)	40(29.8)	Ref	Ref
	Primary	33(49.2)	63(47.0)	0.297	2.61(1.14- 6.60)
	Secondary	19(28.3)	19(14.2)	0.001	5.00(1.91-14.10)
	Tertiary	7(10.4)	11(8.2)	0.061	3.18(0.93-10.92)
County of Residence	Bukonzo East	17(25.3)	37(27.6)	Ref	Ref
	Bukonzo West	20(29.8)	37(27.6)	0.687	1.17(0.53-2.61)
	Bukonzo North	13(19.4)	26(19.4)	0.850	1.08(0.44-2.62)
	Busongora South	16(23.8)	30(22.3)	0.726	1.16(0.50- 2.68)
	Kasese Municipality	1(1.5)	4(2.9)	0.598	0.54(0.026- 4.03)
Household population	>10 people	40(59.7)	75(55.9)	Ref	Ref
	6-10 people	18(26.8)	46(34.3)	0.58	1.29(0.49-3.27)
	1-5 people	9(0.40)	13(9.7)	0.36	0.73(0.37-1.41)

**Environmental water sources**					
Is there a water source within the household?	No	52 (77.61)	110 (82.09)	Ref	Ref
	Yes	15 (22.39)	24 (17.91)	0.45	1.32 (0.34-0.65)

Water source used	Borehole	10 (14.93)	18 (13.43)	0.77	
	River	28 (41.79)	50 (37.31)	0.54	
	Tap (municipal)	46 (68.66)	95 (70.90)	0.74	
	Rainwater Tank	6 (8.96)	17 (12.69)	0.43	
	Unprotected spring	4 (5.97)	16 (11.94)	0.19	
	Protected spring	13 (19.4)	23 (17.16)	0.70	
	Pond	2 (2.99)	3 (2.24)	0.75	
	Shallow well	1 (1.49)	2 (1.49)	1	
	Stream	2 (2.99)	2 (1.49)	0.48	
Is it the water treated at the source?	I don't know	2(2.9)	6(4.5)	Ref	Ref
	Yes	1 (1.54)	19(14.84)	0.16	0.16 (0.007-1.92)
	No	64 (98.46)	109(85.16)	0.50	1.76 (0.39-12.27)
Is it stagnant water?	I don't know	2(2.90)	6(4.51)	Ref	Ref
	Yes	1 (1.54)	6 (4.69)	0.58	1.57(0.35- 0.95)
	No	64 (98.46)	122 (95.31)	0.60	0.50(0.01-6.68)
Is the source shared with animals?	I don't know	2(2.90)	6(4.51)	Ref	Ref
	Yes	20 (30.77)	30 (23.44)	0.42	2 (0.41-14.59)
	No	45 (69.23)	98 (76.56)	0.70	1.38 (0.30-9.65)
Is there direct human access to the water source?	I don't know	2(2.90)	6(4.51)		Ref
	Yes	20 (30.77)	100 (74.63)	0.55	1.67 (0.34-12.11)
	No	45 (69.23)	34 (25.37)	0.65	1.47 (0.32-10.29)

**Individual handling of drinking water in household**					
Is drinking water treated?	Yes	19 (28.36)	30 (11.46)	Ref	Ref
	No	48 (71.64)	104 (77.61)	0.35	1.37 (0.69-2.61)
**Water treatment method**					
Boiling	No			Ref	Ref
	Yes	19 (28.36)	20 (14.93)	0.0252	2.26 (1.10-4.62)
Chlorination	No	59(88.0)	122(91.0)	Ref	Ref
	Yes	8 (11.94)	12 (8.96)	0.51	1.38 (0.52-3.52)
Solar disinfection	No	66(98.5)	134(100)	Ref	Ref
	Yes	1 (1.49)	0	0.99	-
Filtration	No	66(98.5)	133(99.25)	Ref	Ref
	Yes	1 (1.49)	1 (0.75)	0.62	2.01 (0.07-51.49)
Are vessel/ receptacles cleaned on the outside?	No	18 (28.36)	60 (44.78)	Ref	Ref
	Yes	48 (71.64)	73 (55.22)	0.026	2.05 (1.10-3.91)
Is the vessel free of algae?	No	23 (35.82)	72 (53.73)	Ref	Ref
	Yes	43 (64.18)	61 (46.27)	0.017	2.08 (1.14-3.84)
Does the vessel have a cover?	No	36 (55.23)	85 (63.43)	Ref	Ref

	Yes	30 (44.78)	48 (36.59)	0.26	1.41 (0.77-2.55)
Is it sealed with no leakages?	No	4 (7.46)	21 (15.67)	Ref	Ref
	Yes	62 (92.54)	112 (84.33)	0.11	2.30 (0.89-7.17)
Are the water collection vessels clean on the outside?	No	27 (40.30)	74 (55.97)	Ref	Ref
	Yes	40 (59.70)	59 (44.03)	0.037	1.88 (1.04-3.44)
Are the water collection vessels free of algae?	No	46 (68.66)	92 (68.66)	Ref	Ref
	Yes	21 (31.34)	41 (31.34)	1	1 (0.52-1.87)
Do the water collection vessels have covers?	No	60 (89.55)	115 (86.57)	Ref	Ref
	Yes	7 (10.45)	18 (13.43)	0.54	0.15 (0.28-1.83)
Are the water collection vessels free of leakages?	No	5 (7.46)	30 (22.39)		
	Yes	62 (92.54)	104 (77.61)	0.01	3.57 (1.42-10.92)
**Reasons for not treating water**					
	No	61	125	Ref	Ref
Lack of chlorine tablets	Yes	6 (32.84)	9 (17.61)	0.01	2.36 (1.94-4.67)
Believe water is safe for drinking	No	55(82.0)	102(76.1)	Ref	Ref

	Yes	12(18.0)	32(23.9)	0.33	0.69(0.32-1.42)
Lack of firewood*	No	45(67.1)	111(82.8)	Ref	Ref
	Yes	22(32.9)	23(17.2)	0.0133	2.35(1.19- 4.67)
No time to boil water	No	66(98.5)	133(99.3)	Ref	Ref
	Yes	1(1.5)	1(0.7)	0.62	2.01(0.07-51.49)
Water is tasteless when treated	No	57(75.1)	125(93.3)	Ref	Ref
	Yes	10(15.9)	9(6.7)	0.06	2.43(0.93-6.45)
Unavailability of the chlorine tabs	No	65(97.1)	125(93.3)	Ref	Ref
	Yes	2(2.9)	9(6.7)	0.286	0.42(0.06-1.71)
Do you have access to safe water?	No	13 (19.40)	25 (18.66)	Ref	Ref
	Yes	54 (80.60)	109 (81.34)	0.898	0.95 (0.46-2.05)
**Household hygiene**					
Do you wash hands before collecting drinking water?	No	18 (26.87)	34 (25.37)	Ref	Ref
	Yes	49 (73.13)	100 (74.63)	0.82	0.92 (0.48-1.83)
Do wash hands after using the latrine?	No	49 (73.13)	79 (58.96)	Ref	Ref
	Yes	18 (26.87)	55 (41.04)	0.05	0.53 (1.05-4.13)

Do wash hands after urination?	No	46 (68.66)	110 (82.09)	Ref	Ref
	Yes	21 (31.34)	24 (17.91)	0.03	2.09 (1.05-4.13)
Do you was hand with soap and water?	No	19(29.4)	59(45.6)	Ref	Ref
	Yes	48 (71.64)	73 (54.48)	0.02	2.11 (1.14-4.03)
Do you was hand with water only	No	63	123	Ref	Ref
	Yes	4 (5.97)	11 (8.21)	0.57	0.709 (0.19-2.17)
Do you was hand with ash and water	No	65(97.9)	132(98.5)	Ref	Ref
	Yes	2 (2.99)	2 (1.49)	0.48	2.03 (0.24-17.23)
**Personal toilet cleaning**					
Do you use newspapers for cleaning after toilet	No	36(53.7)	87(64.9)	Ref	Ref
	Yes	31(46.3)	47(35.1)	0.126	1.59(0.87-2.90)
Do you use water only for cleaning after toilet	No	48(71.6)	99(73.8)	Ref	Ref
	Yes	19(28.4)	35(26.2)	0.736	1.11(0.57 -2.14)
Do you use water with soap for cleaning after toilet	No	61(91.0)	129(96.3)	Ref	Ref

	Yes	6(9.0)	5(3.73)	0.136	2.53(0.73- 9.11)
Do you use use natural leaves for cleaning after toilet	No	43(64.2)	67(50)	Ref	Ref
	Yes	24(35.8)	67(50)	0.136	0.55(0.30- 1.01)
Do you handle children waste?	No	28 (41.79)	59 (44.03)	Ref	Ref
	Yes	39 (58.21)	75 (55.97)	0.76	1.096 (0.60-1.99)
**Household food safety**					
Do you prepare food on raised ground?	No	40 (59.70)	76 (56.72)	Ref	Ref
	Yes	27 (40.30)	58 (43.28)	0.69	1.13 (0.62-2.06)
Do you ever eat cold leftover food?	No	59 (88.08)	114 (85.07)	Ref	Ref
	Yes	8 (11.94)	20 (14.93)	0.205	0.026 (0.30-1.31)
Do you raw eat vegetables?	No	3 (4.48)	10 (7.52)	Ref	Ref
	Yes	64 (95.52)	123 (93.50)	0.99	-

Do you share food with others in the same container?	No	2 (2.99)	12 (6.97)	Ref	Ref
	Yes	65 (97.01)	122 (91.04)	0.136	3.197 (0.84-20.94
Do animals have access to your food preparation area?	No	44 (65.67)	93 (69.92)	Ref	Ref
	Yes	23 (34.33)	40 (30.08)	0.99	-
Do you own animals?	No	11 (16.42)	34 (25.37)	Ref	Ref
	Yes	56 (83.58)	100 (74.63)	0.15	1.73 (0.84-3.82)
FPA accessed by animals:	No	25 (26.32)	49 (18.48)	Ref	Ref
	Yes	42 (73.68)	85 (82.52)	0.91	0.98 (0.52-1.78)
Do you have barriers physical barriers to your food preparation area?	No	29 (43.28)	59 (44.03)	Ref	Ref
	Yes	38 (56.72)	75 (55.97)	0.42	1.03 (0.62-2.94)
Do you ever find animal droppings in your food preparation area?	No	51 (76.12)	113 (84.33)	Ref	Ref
	Yes	16 (23.88)	21 (15.67)	0.16	1.69 (0.80-3.46)
Found human excrements in food preparation area?	No	64 (95.52)	128 (96.24)	Ref	Ref

	Yes	3 (4.48)	5 (3.76)	0.987	-
**Household sanitation**					
Does this house hold have a latrine?	No	0	4 (2.99)	Ref	Ref
	Yes	67 (100)	130 (97.01)	0.98	-
Is the latrine in use?	No	1 (1.49)	1 (0.77)	Ref	Ref
	Yes	66 (98.51)	129 (99.23)	0.98	-
**Household contamination with Salmonella spp**					
Salmonella spp isolated in house hold environment?	No	51 (79.69)	110 (82.71)	Ref	Ref
	Yes	13 (20.31)	23 (17.29)	0.608	1.22 (0.56-2.57)
Salmonella spp isolated from drinking water	No	58 (86.5)	126 (94.02)	Ref	Ref
	Yes	9 (14.29)	8 (6.15)	0.0804	2.44(0.89-6.82)
Salmonella spp isolated from water source	No	53 (91.38)	108 (89.26)	Ref	Ref
	Yes	5 (8.62)	13 (10.74)	0.659	0.78 (0.24-2.19)
Salmonella spp isolated from of the faecal sample	No	65 (87.50)	128 (77.42)	Ref	Ref

Yes	2 (12.50)	4 (22.58)	0.612	0.65 (0.09-2.94)

*Lack of firewood means lack of any form of fuel i.e. firewood and charcoal (Biofuels)

Table B shows the case predictions from the time series forecasting framework and the empirical date at national and district level for the first quarter of 2017

**Month**	**Week**	**Empirical data**		**Predicted data**		**95%CI**	
Setting		National	Kasese	National	Kasese	National	Kasese
January	1	1850	6	1832.47	9.10	(1557.01-2107.92)	(-0.78 - 18.99)
January	2	1861	13	1803.24	6.64	(1417.70-2188.7)	(-4.25-17.54)
February	4	1165	7	1653.34	5.68	(1182.81-2123.88)	(-6.14-17.51)
February	5	1578	6	1478.72	3.48	(1417.702188.78)	(-9.20-16.17)
February	6	1249	8	1264.34	2.88	(934.35- 2019.10)	(-10.6016.37)
February	7	1391	12	1220.40	3.39	(658.58-1870.09)	(-10.85 - 17.64)
February	8	1631	0	1251.63	5.78	(557.30-1883.50)	(-9.19-20.75)
March	9	1422	8	1187.08	6.71	(535.76 -1967.50)	(-8.94 - 22.37)
March	10	1385	3	1161.18	2.14	(422.8-1952.81)	(-14.173-18.46)
March	11	1739	10	1183.61	2.67	(349.91-1972.25)	(-14.28-19.62)

Text A: Questionnaire used in the case-control study

Questionnaire number: ………… **GPS reading:** ……………………

A: Location details

District: Kasese County: ………..……. Sub-county: ……………

Parish: ………………… Village/LCI: ………………………

B: Demographic details:

B1. Age:…………….

B2. Sex:

1. Female
2. Male

B3. Level of formal education:

1. None
2. Primary
3. Secondary
4. Tertiary

B4. Number of people in the household: ……………………………

B5. Age categories of members:

1. Below 10 years
2. 11-19 years
3. 20-35 years
4. 45-65 years
5. Above 65 years

B5. Position in the household: …………………………

C: Water details

C1. Is the water sourced within the household?

1. Yes
2. No

C2. Where do you get from the water used in this household?

1. Borehole
2. River
3. Lake
4. Tap
5. Rainwater
6. Open spring
7. Protected spring
8. Dug well

C3. What collection vessel do you use to get the water?

1. Jerry can
2. Pot
3. Bucket
4. Basin

C4. Do you have a separate vessel for storage of drinking water?

1. Yes
2. No

C5. If yes, where do you store water meant for drinking?

1. Specified jerry can
2. Pot

C6. Do you treat your drinking water before storage?

1. Yes
2. No

C7. How do you treat this water?

1. Boiling
2. Solar disinfection
3. Chlorination
4. Filtration

C8: If no @ C6, why don't you treat your water?

……………………………………………………………………………………

C8. In case it is a pot, is there a designated cup meant to help retract the water?

1. Yes
2. No

C9. Where do you keep it?

1. In the cupboard
2. On top of the pot
3. On the drying rank
4. Others. Specify: ……………………

C10. Do all family members have free access to this drinking water?

1. Yes
2. No

C11. Do you wash your hands before getting drinking water?

1. Always
2. Sometimes
3. Rarely
4. Not at all

D: Hygiene

D1. Do you wash your hands after visiting the toilet or latrine?

1. Always
2. Sometimes
3. Rarely
4. Not at all

Including urination? ………………..

D2. From where do you wash them?

1. At a designated hand washing facility
2. Using a utensil near the kitchen
3. Direct from water storage vessel

D3. What do you use when washing your hands?

1. With water only
2. With soap and water
3. With ash and water

D4. What method or material do you use for anal cleansing when in the toilet?

1. None
2. Use papers
3. Use toilet paper
4. Use natural anal cleansing leaves
5. Rub against a surface
6. Water only
7. Water and soap

D5. Do you handle children's faecal waste?

1. Yes
2. No

D6. If yes, how do you deal with children's faecal waste?

1. Dump them in the toilet
2. Dump them away from the compound but not in the toilet
3. Just leave them where they are and pour soil/ash

E: Food practises

E1. From where do you prepare food?

1. On raised ground
2. On the ground

E2. How soon do you eat the food you have prepared after preparation?

1. Immediately when still hot
2. Not immediately but still warm
3. Not immediately when it has already cooled

E3. If not immediately, where do you store the food?

1. On the ground
2. In the cabinet
3. On the table
4. In a refrigerator

E4. Do you always preheat the food before consumption?

1. Yes
2. No

E5. Do you usually have left over food?

1. Yes
2. No

E6. How do you deal with leftovers?

1. Eat them
2. Throw them away
3. Feed them to animals

E7. Where do you store the leftovers?

1. On the ground
2. In the cabinet
3. On the table

E8. Do you store your utensils in the kitchen?

1. Yes
2. No

E9. Where do you put the utensils to dry after washing?

1. On the plate stand outside or in the kitchen
2. On the ground in saucepan or basin
3. On the ground on a mat or sack outside

E10. Do you eat any ready to eat foods outside the home?

1. Yes
2. No

E11. Which kind of food?

…………………………………………..

E12. Do you usually have vegetables as part of the diet?

1. Yes
2. No

E13. How do you prepare these before being eaten?

1. Wash them
2. Cook them
3. Wash and/or cook sometimes
4. Just eat them raw without washing or cooking all the time

E14. Do you share the same bowl or plate while eating food especially millet bread?

1. Yes
2. No

E5. Do you have any animals?

1. Yes
2. No

Specify: ………………………………………………………………………………………

E5. Do the animals access the food preparation area?

1. Yes
2. No

Text B: Consent form used in the case control study

Introduction

I am……………………………………………………………..from Makerere University College of Veterinary medicine, Animal resources and Biosecurity. I am carrying out a research about typhoid fever aimed establishing the risk factors that can be related to transmission of the disease within the household and you have been selected as a participant. The burden of typhoid fever in this district is high despite interventions by district health department therefore this study will aim at establishing where the organisms that cause typhoid fever could be sourced in your household and potential practises that could dispose you to them. In order to come up with this information, I will take samples from your drinking water, water source, any food suspected to carry the organisms and fresh human and animal faeces that have potential of contaminating food and water in this household. I will also administer a questionnaire to assess practises related to transmission of typhoid fever. Aside from the valuable time you will offer for this study, the information will never be used against you and will be confidential to purposes only related to this study. The information will not bear your name, only the code that will be awarded to this household. Your participation will not reward you direct benefits however will benefit your community through providing information that can be used in future research or intervention projects. You remain with the right to decline participation in this study or seize your participation at anytime. For more information, please contact the Principal Researcher Mirembe Bernadette Basuta on 0714 145 627 or at Makerere University CoVAB 7062, Kampala. Thank you.

Certificate of consent to the study

I confirm that I have read the information above concerning the study and have had the opportunity to ask questions which were answered adequately. I understand that my participation is voluntary and I am free to withdraw consent at any time, without giving a reason or my legal rights being affected. I understand that data collected can be used for purposes of monitoring and auditing by the University officials to ensure that the study is being conducted properly. I consent to access of relevant information needed and I accept to participate in this study.

Name: ……………………………………… Signature: ………………………
